# The characterization of RNA-binding proteins and RNA metabolism-related proteins in fungal extracellular vesicles

**DOI:** 10.3389/fcimb.2023.1247329

**Published:** 2023-09-14

**Authors:** Marianna Dallastella, Willian Klassen de Oliveira, Marcio L. Rodrigues, Samuel Goldenberg, Lysangela R. Alves

**Affiliations:** ^1^ Gene Expression Regulation Laboratory, Carlos Chagas Institute (ICC), Oswaldo Cruz Foundation, FIOCRUZ, Curitiba, Brazil; ^2^ Laboratory for Applied Sciences and Technology in Health, Carlos Chagas Institute, FIOCRUZ PR, Curitiba, Brazil; ^3^ Microbiology Institute, Federal University of Rio de Janeiro (UFRJ), Rio de Janeiro, Brazil; ^4^ Research Center in Infectious Diseases, Division of Infectious Disease and Immunity CHU de Quebec Research Center, University Laval, Quebec, QC, Canada

**Keywords:** extracellular vesicles, RNA-Binding Proteins, RNA metabolism, proteomic data, cellular communication

## Abstract

RNA-binding proteins (RBPs) are essential for regulating RNA metabolism, stability, and translation within cells. Recent studies have shown that RBPs are not restricted to intracellular functions and can be found in extracellular vesicles (EVs) in different mammalian cells. EVs released by fungi contain a variety of proteins involved in RNA metabolism. These include RNA helicases, which play essential roles in RNA synthesis, folding, and degradation. Aminoacyl-tRNA synthetases, responsible for acetylating tRNA molecules, are also enriched in EVs, suggesting a possible link between these enzymes and tRNA fragments detected in EVs. Proteins with canonical RNA-binding domains interact with proteins and RNA, such as the RNA Recognition Motif (RRM), Zinc finger, and hnRNP K-homology (KH) domains. Polyadenylate-binding protein (PABP) plays a critical role in the regulation of gene expression by binding the poly(A) tail of messenger RNA (mRNA) and facilitating its translation, stability, and localization, making it a key factor in post-transcriptional control of gene expression. The presence of proteins related to the RNA life cycle in EVs from different fungal species suggests a conserved mechanism of EV cargo packing. Various models have been proposed for selecting RNA molecules for release into EVs. Still, the actual loading processes are unknown, and further molecular characterization of these proteins may provide insight into the mechanism of RNA sorting into EVs. This work reviews the current knowledge of RBPs and proteins related to RNA metabolism in EVs derived from distinct fungi species, and presents an analysis of proteomic datasets through GO term and orthology analysis, Our investigation identified orthologous proteins in fungal EVs on different fungal species.

## Introduction

Cell-to-cell communication involves distinct pathways, such as direct contact, secretion of diverse molecules, and the transfer of information through extracellular vesicles (EVs) (Raposo and Stahl, 2019). EVs are particles limited by a double lipid layer, unable to replicate, and able to carry several functional molecules (Tkach and Théry, 2016). EVs have been described in all domains of life, from prokaryotes to mammals and plants (Yáñez-Mó et al., 2015; Munhoz da Rocha et al., 2020). There are two main groups of EVs: microvesicles that bud out of the plasma membrane and exosomes that originate from the endosomal pathway within multivesicular bodies (Yáñez-Mó et al., 2015; Raposo and Stahl, 2019). The apoptotic bodies consist on another type of EV, that are derived from the disassembly cell undergoing apoptosis (Yáñez-Mó et al., 2015; Raposo and Stahl, 2019).

The main functions of EVs related to cellular communication include the transfer of molecules, changing gene expression patterns, and surface rearrangements (Fang et al., 2022). In addition, they can also deliver virulence factors, act in the antigen presentation process, stimulate an immune response or tolerogenic effect, and promote immunosuppression, angiogenesis, and tumor progression (van Niel et al., 2018). In some microorganisms, EV secretion is also used to promote microbial survival and pathogenesis (Munhoz da Rocha et al., 2020). Pathogens utilize EV traffic to manipulate the host, induce the recruitment of specific immune cells, and contribute to their life cycle and reproduction (Dong et al., 2019).

A remarkably diverse array of proteins has been identified in the EVs of different fungal species. For example, the most abundant proteins identified in four fungi species are the elongation factor 1 alpha, nuclear proteins such as Histone H4.2, and other proteins related to stress response, carbohydrate, lipid, and protein metabolism (Rodrigues et al., 2011; Rodrigues et al., 2014; Vallejo et al., 2012). The most ubiquitous ortholog proteins present in all species studied to date are the heat shock Hsp70 protein (PF00012), a chaperone-encoding gene, the nucleoside diphosphate kinase, and the ribosomal S17 protein (Parreira et al., 2021).

EVs containing RNA molecules were first described in murine and human cells in 2007 raising the assumption that these structures participate in cell-to-cell communication and modulate protein expression in recipient cells (Valadi et al., 2007).

Fungal EVs from ascomycetes and basidiomycetes contain a plethora of molecules, including proteins, glycans, lipids, and nucleic acids (Rodrigues et al., 2007; Albuquerque et al., 2008). RNA molecules have been characterized in diverse fungal species, such as *Cryptococcus neoformans*, *Candida albicans*, *Paracoccidiodes brasiliensis*, *Saccharomyces cerevisiae*, *Malassezia sympodialis*, and *Histoplasma capsulatum* (Peres da Silva et al., 2015; Rayner et al., 2017; Alves et al., 2019). Recently, the interaction between diverse plant species and pathogens has been studied, and EV’s containing different cargoes, including RNA molecules (Kwon et al., 2021; He et al., 2023), have been described (Bleackley et al., 2020; Hill and Solomon, 2020). The RNAs described in fungal EVs include messenger RNA (mRNAs), transfer RNA (tRNAs), ribosomal RNA (rRNAs), and small non-coding RNAs (sncRNAs), and these RNA types play essential roles in post-transcriptional regulation, protein translation, RNA processing, and stability (Turchinovich et al., 2019).

Addressing molecules for loading into the EVs is a regulated process that responds to disruptions in cellular homeostasis, but the mechanisms involved are poorly understood, especially regarding RNA cargo. Recent studies in mammalian cell models and also plant models have hypothesized that sorting and addressing RNA molecules to EVs involve RNA-binding proteins (Santangelo et al., 2016; Fabbiano et al., 2020; Groot and Lee, 2020; He et al., 2021). Some authors have found evidence that link the sorting of RNA molecules towards EV’s with RNA binding proteins, such as: the Heterogeneous nuclear ribonucleoprotein A2B1 (Villarroya-Beltri et al., 2013); Argonaute 2 protein (McKenzie et al., 2016), the Major Vault Protein (Teng et al., 2017).

To shed light on EV biology, this review aims to extensively characterize the EV proteomic datasets derived from fungi to identify RNA binding proteins and other proteins involved in RNA metabolism.

### Pathogenic fungi

Fungal diseases affect nearly a billion people worldwide, ranging from hair or nail infections to highly lethal systemic fungal diseases. A plasma membrane and a complex cell wall structure delimit fungal cells. Despite this additional barrier, these cells can secrete extracellular vesicles (Rodrigues et al., 2016). Most fungal diseases affect both immunocompromised and immunocompetent individuals, though morbidity and mortality typically markedly increase in the setting of immune dysfunction. Examples are *H. capsulatum*, which causes fungal pneumonia (Allen and Deepe, 2005); *C. neoformans* and *Cryptococcus deuterogattii*, which cause cryptococcosis, a meningoencephalitis (Perfect and Casadevall, 2002); *Aspergillus fumigatus*, which causes invasive pulmonary infection or aspergillosis (Patterson et al., 2000); and *C. albicans*, which can cause superficial mucosal or dermal infections, as well as disseminated candidiasis (Pfaller and Diekema, 2007; Kullberg and Arendrup, 2015). In addition, *Sporothrix schenkii* and *Sporothrix brasiliensis* cause sporotrichosis in felines and humans, leading to lesions on the skin and subcutaneous cellular tissue (Barros et al., 2011; Rodrigues et al., 2020).

Besides affecting humans, pathogenic fungi can cause disease on different plant species (Li et al., 2020). The *Fusarium oxysporum* f. sp. *vasinfectum*. is a pathogen that affects cotton leaves causing phytotoxic response (Bleackley et al., 2019); *Zymoseptoria tritici* affects wheat (Hill and Solomon, 2020) and *Ustilago maydis* affects maize (Kwon et al., 2021), both plant-pathogens causing yield reductions in crops (Hill and Solomon, 2020; Kwon et al., 2021).

### RNA metabolism

RNA-binding proteins (RBPs) are essential in cellular processes involved in RNA metabolism, from transcription to decay. During transcription, RBPs associate with RNA to protect it from degradation and regulate its fate in the cell. RNA processing, including splicing, alternative splicing, and trans-splicing, relies on the proper recognition and exposure of RNA sequences to the splicing machinery (Lee and Rio, 2015). After processing, mature mRNAs are transported to the cytoplasm (Björk and Wieslander, 2017),, directed to translation or stored in RNA granules for silencing or degradation. RBPs associate with mRNAs and assemble in ribonucleoprotein complexes (mRNPs) and mediate RNA processing, transport, and localization within the cell, thereby determining mRNA fate based on the composition of each mRNP complex (Gerstberger et al., 2014; Re et al., 2014).

The arrangement and assembly of RBPs in mRNP complexes are highly dynamic (Müller-McNicoll et al., 2016; Buchan, 2014). Different sets of RBPs associate with mRNA to enable its functionality. The combinatorial arrangement of proteins onto a given mRNP complex remains elusive and is a significant challenge for researchers. In addition to the genetic and histone code, an RBP code may exist, given the number of known RBPs and proteins that function as RBPs. This code is complex and ultimately determines gene expression.

Numerous studies and databases describe the presence of RBPs in EVs of different organisms, including pathogens (Vesiclepedia [http://microvesicles.org]; (Kalra et al., 2012; Pathan et al., 2019). This analysis of fungal RBPs is based on an extensive analysis of EV proteomic data publicly available.

## Results

### Proteomic data

We systematically analyzed available proteomic datasets aiming to identify proteins involved in RNA metabolism present in fungal EVs. The species with available proteomic data were *C. albicans, C. neoformans, C. deuterogattii, H. caspsulatum, P. brasiliensis, S. cerevisiae, A. fumigatus, S. brasiliensis*, and *S. schenckii*. The most applied method to isolate fungal EVs has been through differential centrifugation followed by filtration with porosity limits varying from 0.45 μm to 1.2 μm (Albuquerque et al., 2008; Rodrigues et al., 2008; Oliveira et al., 2010; Vallejo et al., 2012; Wolf et al., 2014; Gil-Bona et al., 2015; Vargas et al., 2015; Wolf et al., 2014; Matos Baltazar et al., 2016; Ikeda et al., 2018; Zarnowski et al., 2018; Rizzo et al., 2020a; Rizzo et al., 2020b). This method allows for isolating a broader range of EVs with different sizes (**Supplemental Table 1**).

**Figure 1:**
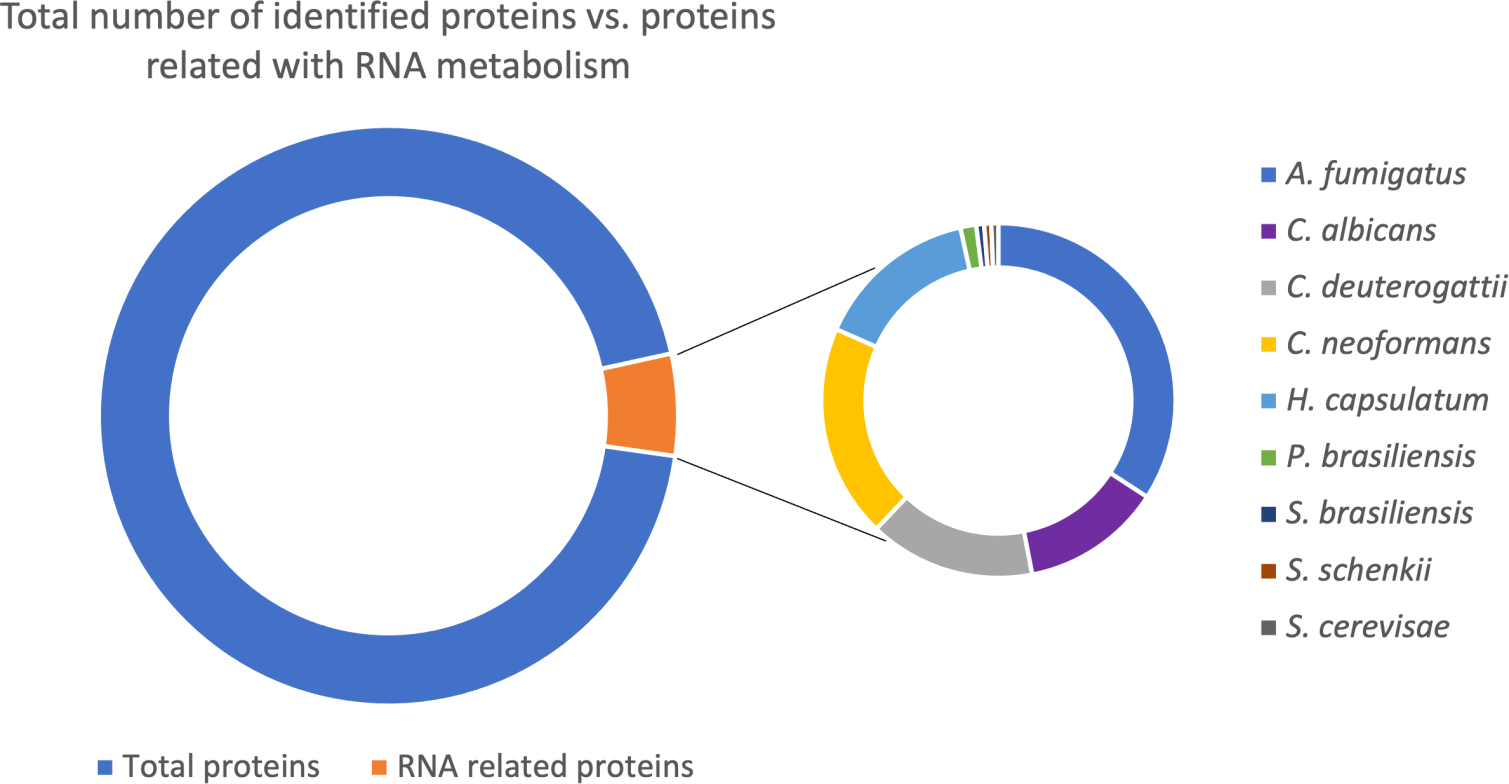
Pie chart with the total number of proteins in fungal EVs identified (11433 proteins); from this amount, the proteins related to RNA metabolism (687 proteins). The second graph is related to the 687 proteins RNA related divided by species in which the proteins were identified.

Based on the available EV proteomic datasets (Parreira et al., 2021), we identified that around 7% of the total proteins in fungal EVs relate to RNA metabolism (**Figure 1**; **Supplemental Table 2**). Furthermore, after removing the redundancy of the distinct strains from the same species, we identified 687 proteins associated with different steps of RNA metabolism, which we discuss in detail in this work.

The RNA life cycle is highly regulated and dynamic, with each step playing a critical role in determining the fate, functionality, and abundance of RNA molecules within a cell. In the datasets we analyzed, proteins involved in various processes that regulate the RNA molecules were identified in the EVs.

Among the proteins in EVs related to transcription, Sm-like proteins were observed. Proteins belonging to this family can form small nuclear ribonucleoprotein complexes (snRNPs) by assembling with snRNAs during nuclear mRNA splicing, the process of removing introns from pre-mRNA. Various complexes can be formed depending on the protein family bound to the snRNA molecules (Will and Luhrmann, 2011). As snRNAs and snoRNAs are significantly enriched in EVs, the presence of Sm-like proteins reinforces the important role of these snRNP complexes in the EVs (**Table 1**).

**Table 1:** List of Sm-like proteins found on extracellular vesicles from fungi species.

Protein name	*C. neoformans*	*C. deuterogattii*	*A. fumigatus*	*H. capsulatum*
U6 snRNA-associated Sm-like protein LSm1		•	•	
U6 snRNA-associated Sm-like protein LSm2	•	•		•
U6 snRNA-associated Sm-like protein LSm3	•	•		•
U6 snRNA-associated Sm-like protein LSm4	•	•	•	
U6 snRNA-associated Sm-like protein LSm5	•	•	•	
U6 snRNA-associated Sm-like protein LSm6	•	•		
U6 snRNA-associated Sm-like protein LSm7	•	•		
U3 small nucleolar RNA-associated protein 22			•	

Translation initiation factors are a group of proteins crucial for initiating protein synthesis. EVs derived from human and mouse cells also contain translation initiation factors, such as eIF4E and eIF4G, and some are used as biomarkers for certain cancer types (Dong et al., 2020). We also identified eukaryotic translation initiation factors (eIFs) in EVs from different fungal species. We observed the presence of eIF3H, eIF5A, eIF2A, and eIF6 in most of the fungal EVs species we analyzed (**Table 2**). This finding is consistent with other proteomic studies in various cell models where many eIFs have been identified (Kalra et al., 2012; Pathan et al., 2019).

**Table 2:** List of translation initiation factors identified in fungal EVs.

	*A. fumigatus*	*C. albicans*	*C. deuterogattii*	*C. neoformans*	*H. capsulatum*
Recognition of the mRNA cap structure: eIF4F complex					
Eukaryotic translation initiation factor eIF-4A	•				
Eukaryotic translation initiation factor 4G		•	•	•	
Eukaryotic translation initiation factor 4E	•	•		•	
Recruitment of the ribosome to the mRNA					
Eukaryotic translation initiation factor eIF-1A	•	•			
Eukaryotic translation initiation factor 3 subunit A	•	•		•	
Eukaryotic translation initiation factor 3 subunit B	•				
Eukaryotic translation initiation factor 3 subunit C	•		•	•	•
Eukaryotic translation initiation factor 3 subunit D	•			•	•
Eukaryotic translation initiation factor 3 subunit E	•				
Eukaryotic translation initiation factor 3 subunit F	•		•	•	•
Eukaryotic translation initiation factor 3 subunit G	•		•	•	
Eukaryotic translation initiation factor 3 subunit H	•	•	•	•	•
Eukaryotic translation initiation factor 3 subunit I	•			•	•
Eukaryotic translation initiation factor 3 subunit J	•		•	•	
Eukaryotic translation initiation factor 3 subunit K	•		•	•	
Eukaryotic translation initiation factor 3 subunit L	•			•	•
Eukaryotic translation initiation factor 3 subunit M	•		•	•	•
Eukaryotic translation initiation factor 5A	•	•	•	•	•
Joining of the initiator tRNA to the mRNA					
Eukaryotic translation initiation factor 2A	•	•	•	•	•
Translation initiation factor eIF-2B alpha subunit	•	•			
Translation initiation factor eiF-2B delta subunit	•	•			
Translation initiation factor eIF-2B epsilon subunit	•	•			
Dissociation of initiation factors and ribosome scanning					
Translation initiation factor 4B	•		•	•	
Translation initiation factor eIF5		•			
80S formation regulation					
Eukaryotic translation initiation factor 6	•	•	•	•	•

Another group of proteins related to translation identified in the EVs are elongation factors 1 (EF1) and 2 (EF2). They are vital proteins involved in the elongation phase of translation. EF2 is a very abundant protein in the cell and is also the top protein identified in EVs derived from proteomic studies in distinct species (Kalra et al., 2012; Pathan et al., 2019).

Helicases are another class of proteins involved in RNA metabolism (Linder and Jankowsky, 2011). The DEAD/DEAH protein family, found in all kingdoms, is the largest family of RNA helicases and is involved in various RNA metabolic steps such as RNA synthesis and folding, RNA-RNA interactions, RNA degradation, and localization (Gilman et al., 2017). Several RNA helicases have been described in fungal EVs, listed in **Table 3**.

**Table 3:** List of helicase proteins found on extracellular vesicles from fungi species.

Pathogen	Protein type	Proteins names			
*C. neoformans*	DEAD/DEAH box ATP-dependent RNA helicase	DBP5	DED1		
	ATP-dependent RNA helicase	SUB2	FAL1	DBP10	
*C. deuterogattii*	DEAD/DEAH box ATP-dependent RNA helicase	DBP5	DED1	DHH1	
	ATP-dependent RNA helicase	SUB2	EIF4A		
*C. albicans*	DEAD/DEAH box ATP-dependent RNA helicase	DBP5			
	ATP-dependent RNA helicase	SUB2	FAL1		
*H. capsulatum*	DEAD/DEAH box ATP-dependent RNA helicase	DBP5	DED1	DHH1	
	ATP-dependent RNA helicase	SUB2	FAL1		
*A. fumigatus*	DEAD/DEAH box ATP-dependent RNA helicase	DBP5	DHH1	ROK1	DBP9
	ATP-dependent RNA helicase	SUB2	DSR1	PRP5	
*S. brasiliensis*	ATP-dependent RNA helicase	MAK5			

### RNA-binding proteins

RNA-binding proteins are essential in most, if not all, stages of the RNA life cycle. Distinct structural features and RNA-binding mechanisms characterize several types of RNA-binding domains. The RRM domain is the most common RNA binding domain, with each domain being specific for RNA sequences and interacting with single-stranded RNA molecules. Proteins with RRM domains are involved in all steps of RNA metabolism. Many fungal species have RNA-binding proteins with RRM domains enclosed in EVs, as listed in **Table 4**. One example is the polyadenylate-binding protein (PABP), a conserved protein that plays a crucial role in RNA metabolism.

**Table 4:** List of RNA binding proteins divided by domains found in the different fungal EVs.

	Protein	Pathogen
**RRM domain**	Glycine-rich RNA binding protein	*C. neoformans*
	RNA binding protein (J9VH85)	
	RNA-binding protein Musashi	
	RNA-binding protein with a serine-rich domain	
	Pre-mRNA branch site protein p14	
	Pre-mRNA-splicing factor SLT11	
	mRNA binding protein (A0A095CI41)	*C. deuterogattii*
	RNA binding protein (A0A095ES51)	
	RNA binding protein (A0A095CIW0)	
	glycine-rich RNA binding protein	
	RNA-binding protein (A0A1D8PK11)	*C. albicans*
	RNA binding protein Rnp24	*S. schenckii*
	mRNA binding post-transcriptional regulator	*A. fumigatus*
	Pre-mRNA splicing factor	
	RNA binding proteins	
	Nuclear and cytoplasmic polyadenylated RNA-binding protein pub1	*H. capsulatum*
	RNA binding domain-containing protein (C0NSY4)	
	RNA binding domain-containing protein (C0P155)	
	RNA binding protein (C0NB22)	
	Polyadenylate-binding protein pub1	*A. fumigatus*
	Polyadenylate-binding protein pub1	*H. capsulatum*
**Zinc finger domain**	RNA binding protein containing a zinc finger	*A. fumigatus*
	RNA polymerase II transcription factor	*C. neoformans*
	RNA polymerase II transcription factor	*C. deuterogattii*
**KH domain**	KH domain RNA binding proteins C0NUH0	*H. capsulatum*
	KH domain RNA binding proteins C0NCT3	
	KH domain RNA binding protein B0XU88	*A. fumigatus*
	KH domain RNA binding protein B0XVE5	

Zinc finger proteins constitute a large family of proteins primarily associated with DNA binding but also capable of binding to RNA and small molecules, such as the CCCH zinc finger proteins involved in regulating RNA metabolism. Proteomics data in **Table 4** lists zinc finger domain-containing proteins found in EVs derived from the pathogens reviewed in this study, with functions related to RNA binding, RNA metabolic processes, and rRNA binding. The RNA polymerase II transcription factor contains a zinc finger RNA binding domain. This protein modulates DNA-templated transcription and participates in the assembly of RNA polymerase II preinitiation complex, forming the first bonds in the RNA chain.

The hnRNP K-homology (KH) domain is one of the most prevalent RNA binding domains and is found in proteins responsible for regulating gene expression in prokaryotes and eukaryotes. In addition, this domain binds specific sequences on nucleic acids (**Table 4**).

### GO term analysis

**Figure 2:**
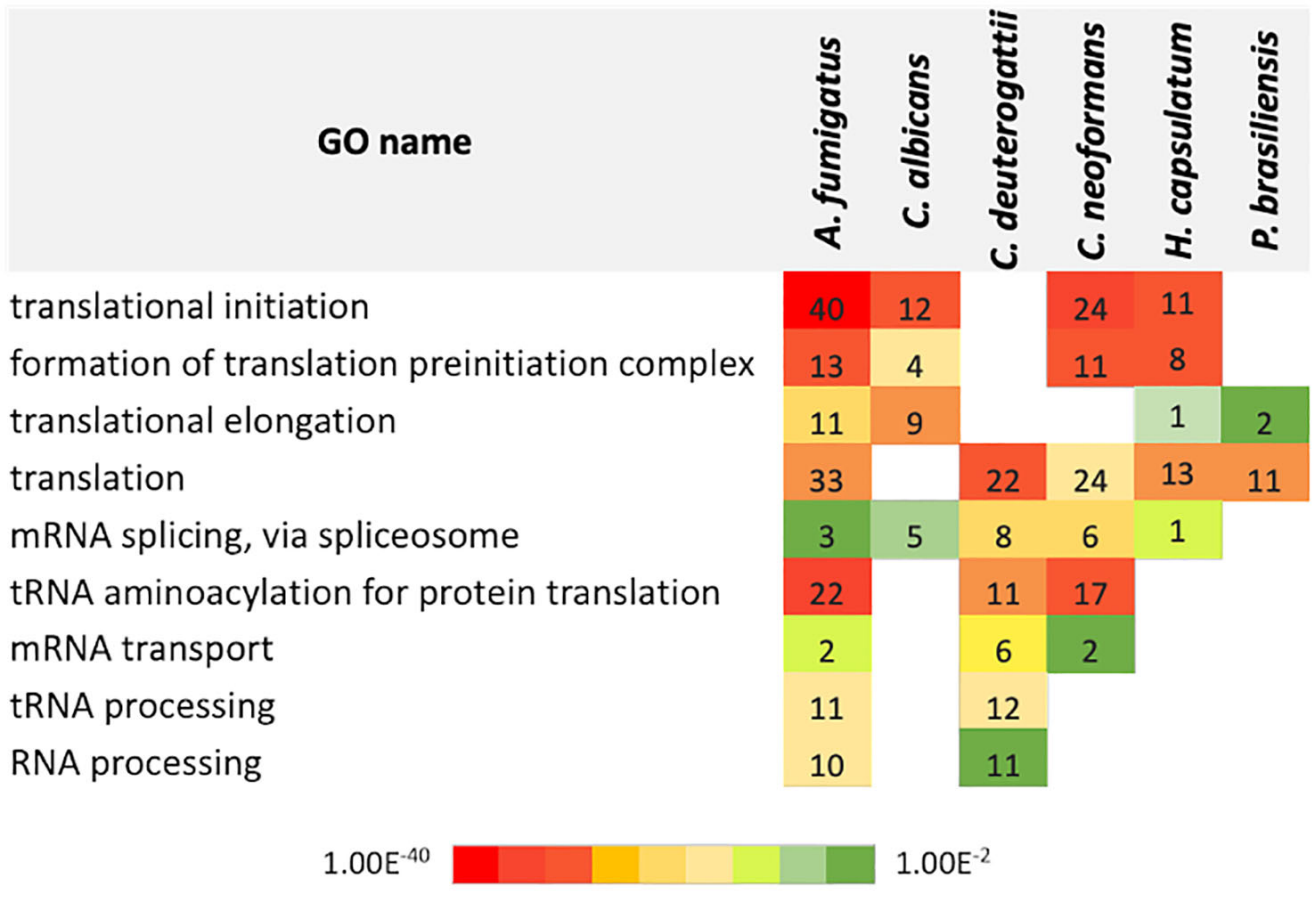
Chart with the gene ontology terms related to biological processes in EVs identified for each fungal species. The numbers refer to the total count of proteins for each category. The color code refers to the adjusted p-value of Fisher’s exact test.

To determine the RNA pathways enriched in fungal EVs and to compare them among the species, we performed a gene ontology (GO) term analysis with each proteomic dataset (**Figure 2**). Proteins related to translation were the most prominent category, followed by proteins functioning as translation initiation factors and proteins involved in translation elongation. Interestingly, the subsets showed no enrichment of proteins involved in non-coding RNA (ncRNA) biosynthesis or processing machinery. However, most RNA molecules present in the EVs belong to the ncRNA class (Peres da Silva et al., 2015).

### Orthology analysis

**Figure 3:**
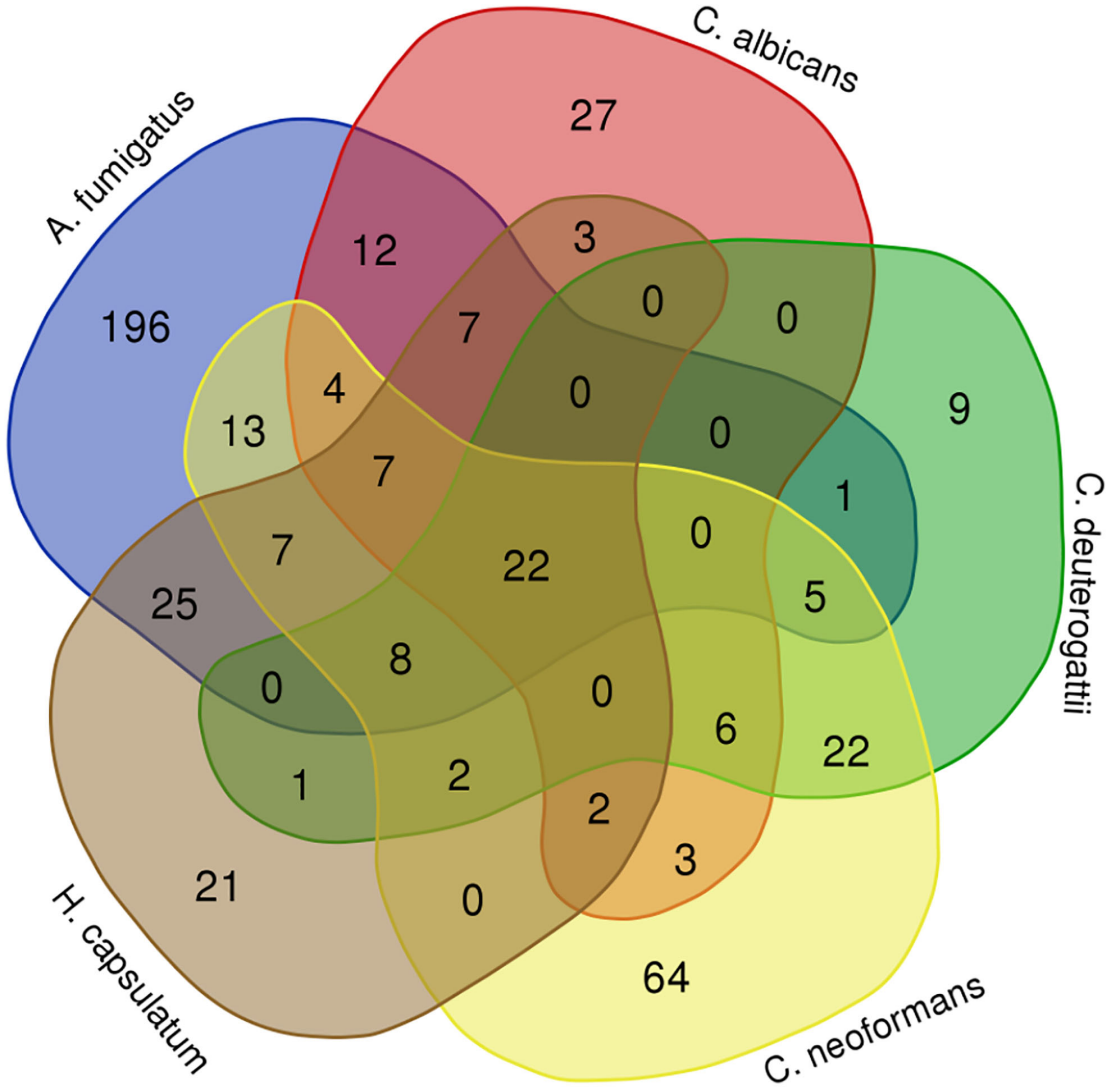
Orthology analysis. The Venn diagram illustrates the number of orthologous proteins identified in the EVs of the analyzed fungal species. Each overlapping region represents proteins shared between the respective species. The 22 common proteins are highlighted in green in the **Table S2**.

Next, we conducted a comparative analysis of the proteins present in the EVs of various fungal species, aiming to identify orthologs. Through this analysis, we observed the formation of forty-nine protein clusters, each comprising at least three species (**Supplemental Table 3**). A total of 22 proteins were present in at least five species studied (**Figure 3**). Among the proteins with the highest number of orthologs, we identified: the Elongation factor 1-alpha, with a total of 10 orthologs in *C. albicans, H. capsulatum, C. neoformans, C. deuterogattii, P. brasiliensis*, and *A. fumigatus*, with at least 84% similarity among them; the ATP-dependent RNA helicase eIF4A/Helicase FAL1 with eight orthologs in *H. capsulatum, C. neoformans, C. deuterogattii, C. albicans*, and *A. fumigatus*, with at least 68% similarity; and Elongation Factor 2 with seven orthologs in *C. albicans, A. fumigatus, C. neoformans, C. deuterogattii, H. capsulatum*, and *P. brasiliensis*, with at least 75% similarity among the proteins. The top clusters are listed in **Table 5**.

**Table 5:** Top protein clusters identified in the fungal EVs.

Uniprot ID	Similarity	Species	Uniprot ID	Similarity	Species
Elongation factor 1-alpha			Seryl-tRNA synthetase		
B0XPK2	*	*A. fumigatus*	B0Y360	68.14%	*A. fumigatus*
C4YDJ3	88.43%	*C. albicans*	Q9HGT6	57.79%	*C. albicans*
A0A095DDT6	85.19%	*C. deuterogattii*	A0A095DHL3	50.11%	*C. deuterogattii*
J9W2J0	84.78%	*C. neoformans*	J9VKD0	50.33%	*C. neoformans*
A6RGN1	89.57%	*H. capsulatum*	C0NE91	*	*H. capsulatum*
C1G1F2	89.13%	*P. brasiliensis*			
ATP-dependent RNA helicase eIF4A/Helicase FAL1			Isoleucyl-tRNA synthetase		
B0XYH6	*	*A. fumigatus*	B0XRM5	55.64%	*A. fumigatus*
P87206	70.78%	*C. albicans*	Q59RI1	55.61%	*C. albicans*
A0A095ECV2	72.57%	*C. deuterogattii*	A0A095CYT6	*	*C. deuterogattii*
J9VHS2	72.57%	*C. neoformans*	J9VDL3	98.08%	*C. neoformans*
A6R3R5	90.91%	*H. capsulatum*	C0NL66	53.89%	*H. capsulatum*
Eukaryotic translation initiation factor 3 subunit I			Polyadenylate-binding protein, PABP		
B0XYC8	60.88%	*A. fumigatus*	B0XND2	68.39%	*A. fumigatus*
Q5AI86	*	*C. albicans*	A6RAN8	82.24%	*C. albicans*
J9VEG9	50.15%	*C. deuterogattii*	C0NSS5	88.27%	*C. deuterogattii*
A6R3Z8	59.71%	*C. neoformans*	C1GL98	*	*C. neoformans*
C0NAW2	59.71%	*H. capsulatum*	A0A0C2FSY1	58.99%	*H. capsulatum*
ATP-dependent RNA helicase DED1			ATP-dependent RNA helicase DHH1		
B0Y5V9	67.21%	*A. fumigatus*	B0XZ91	65.29%	*A. fumigatus*
Q5A4E2	54.02%	*C. albicans*	Q5AAW3	62.66%	*C. albicans*
A0A095C6F2	58.45%	*C. deuterogattii*	A0A095CDG4	92.52%	*C. deuterogattii*
J9VMP7	58.24%	*C. neoformans*	Q58Z64	*	*C. neoformans*
C0NAF4	*	*H. capsulatum*	C0NIX7	64.77%	*H. capsulatum*

The relevant finding is that many proteins participating in different steps of RNA metabolism were conserved among other fungal species, such as eukaryotic translation initiation factors, helicases, elongation factors, and tRNA synthetases. Among the proteins containing known RNA binding domains, we observed the Polyadenylate-binding protein (PAB1), RRM-containing protein, KH domain RNA-binding protein, and nuclear and cytoplasmic polyadenylated RNA-binding protein (pub1).

**Figure 4:**
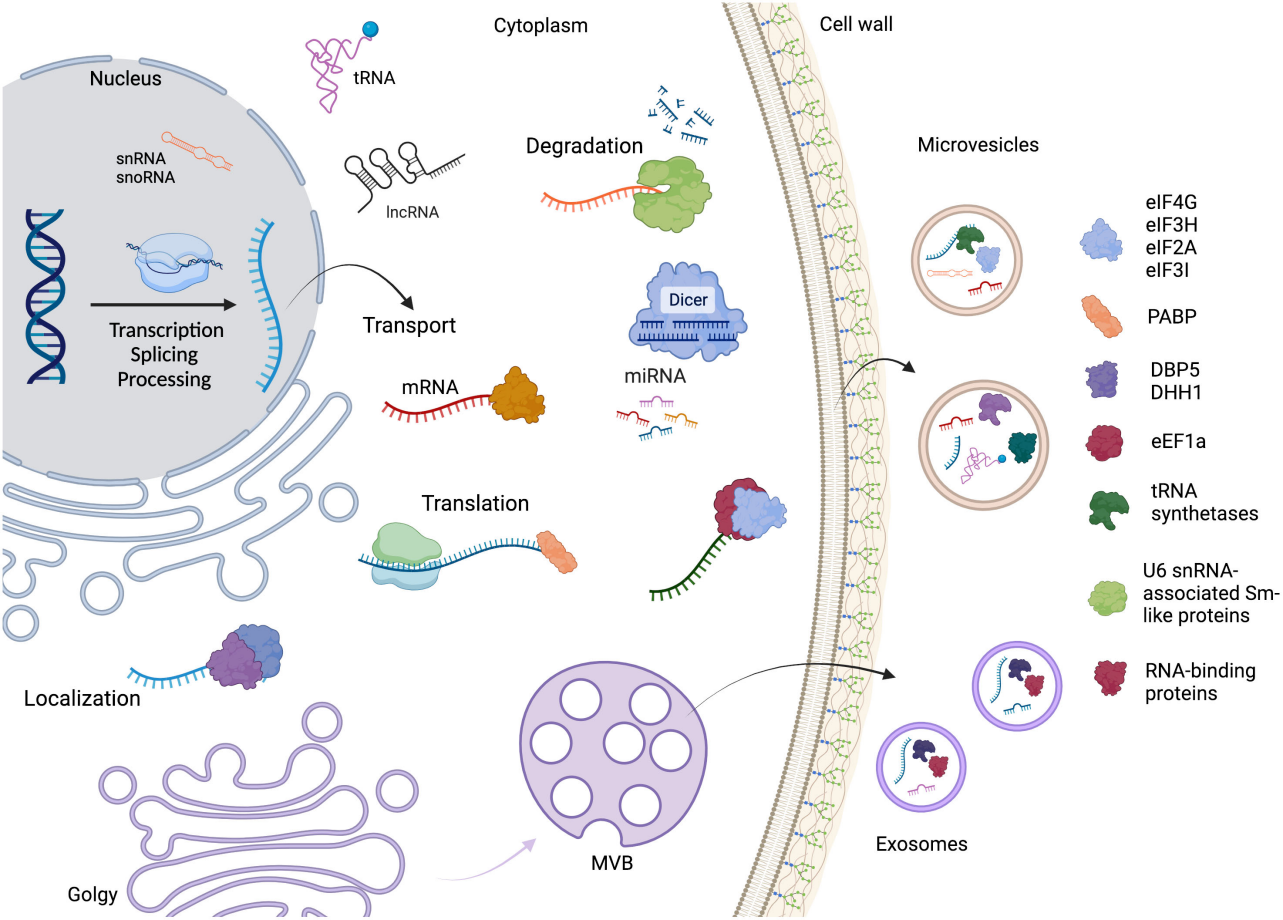
The RNA life cycle encompasses various stages, including transcription, processing, and degradation. Within this cycle are several distinct classes of RNA molecules, such as mRNA, miRNA, snoRNA, snRNA, tRNA, and lncRNA. Throughout RNA metabolism, different RNA-binding proteins play crucial roles in various steps. Additionally, the mechanism by which RNAs and proteins are directed to extracellular vesicles (EVs) remains unknown. However, our study focused on identifying and highlighting the key proteins found in fungal EVs. Created with BioRender.com.

These observations reinforce the hypothesis that RBPs, or even mRNPs, drive the selection and direction of RNA to the EVs, as many of them are conserved in phylogenetically distant fungal species. A summary of the RNA life cycle, the main proteins identified in the EVs, and the connection between the RNA metabolism and extracellular vesicles is depicted in **Figure 4**.

## Discussion

The biogenesis of EVs is complex and involves distinct pathways. Exosomes are produced in endosomes and later secreted upon fusion with the cell surface, while microvesicles are formed by outward budding from the plasma membrane (van Niel et al., 2018). In both EV types, the cargo is transferred or displayed to target cells, causing a specific biological effect (Sork et al., 2018). The mechanism by which the EVs cargo is selected remains unclear. However, recent research has promoted advances in knowledge, such as the description that proteins associated with autophagy (ATG8) and microtubule (LC3) form a complex LC3/ATG8, which can mediate the loading of protein and RNA into EVs of HEK-293T cells (Gardner et al., 2023). Furthermore, other studies suggested the association between RNA-binding proteins and EVs through the formation of complexes to transport the RNA molecules (Statello et al., 2018; Anand et al., 2019; Fabbiano et al., 2020). In addition, the RNA binding proteins hnRNPA2B1, Ago2, YBX-1, MEX3C, MVP, and La may participate in the selection and transfer of miRNA into EVs (Groot and Lee, 2020).

However, this literature primarily focuses on mammalian EVs, and our knowledge of RNA associations with fungal EVs is limited. Our orthology analysis reveals that conserved proteins participate in RNA metabolism among different fungi species. For example, two of the proteins with the highest number of orthologous matches among the top 100 most identified proteins are the eukaryotic translation elongation factor 1 alpha 1 (EEF1A1) and elongation factor 2. The presence of full-length mRNA transcript on exosomes-like vesicles of Toxoplasma-infected cells has been observed, and among the most highly represented mRNA was the EEF1A1 (Pope and Lässer, 2013). This protein has also been previously found on exosomes secreted by adipose-derived stem cells (Kuo et al., 2021); and on small EVs isolated from patients with non-small cell lung cancer (Bui et al., 2022).

Among the proteins found in our analysis, there were many ribosomal proteins, which are a variety of proteins that compose the ribosome and participate in the folding of rRNA molecules into a structure that is required for the interaction of mRNA codons and tRNA anticodons (Landry-Voyer et al., 2023). We excluded this class of proteins to avoid bias toward translation.

RNA helicases are regulators of multiple pathways of RNA metabolism (Sloan and Bohnsack, 2018). DEAD-box helicases are on EVs of the plant *Arabidopsis*, among other RNA-binding proteins contributing to the sRNA loading into EVs (He et al., 2021). Interestingly, the DEAD-box RNA helicase Dbp5, which is a critical mRNA export factor, shuttling between the nucleus and cytoplasm (Bleackley et al., 2019; Lari et al., 2019), was detected in EVs in four of the fungi we analyzed. The RNA helicase DHH1 (DDX6 in humans) is an essential protein for RNA granule formation, including processing bodies (p-bodies) and stress granules. These structures are formed by dozens of proteins interacting with mRNA and assembled under certain conditions to store or degrade RNA (Decker and Parker, 2012; Riggs et al., 2020). DHH1, a core nucleator of these granules, was identified in EVs from mammalian cells. A recent study showed a possible link between p-bodies and EV cargo as the RNA-binding protein YBX1 was necessary to direct miR-223 to EVs with YBX1 associating with p-bodies and colocalizing with DHH1 in the cell, and both are found in EVs. Therefore, it is possible to speculate that p-bodies could work as a site to sort the RNA-protein cargo directed to EVs (Liu et al., 2021).

Another group of enriched proteins in EVs are the aminoacyl-tRNA synthetases, enzymes responsible for the acetylation of tRNA molecules by the cognate amino acid, participating in the first step of protein synthesis (Mirande, 2017). Besides their primary function, they also support RNA splicing, regulate transcription and translation, and participate in cell signaling (Pang et al., 2014). Interestingly, tRNA fragments are among the most common RNA molecules detected in EVs in all organisms characterized to date (Peres da Silva et al., 2015; Weng et al., 2022). Therefore, it is possible to speculate that the tRNA-half fragments are associated with the aminoacyl-tRNA synthetases and addressed to EVs. Aminoacyl-tRNA synthetases are also enriched in the exosomes derived from Jurkat cells (Wang et al., 2013). The aminoacyl-tRNA synthetases: leucyl-, isoleucyl-, arginyl- and valyl-tRNA synthetases were found with glycyl-tRNA synthetase 1 (GARS1) in EV derived from macrophages, under glucose starvation (Goughnour et al., 2020). Additionally, GARS1 is secreted *via* specific EVs and promotes cancer cell apoptosis. These GARS1-containing EVs, enriched with unique proteins, including insulin-like growth factor II receptor and vimentin, contribute to the immunological defense against tumorigenesis (Goughnour et al., 2020). Cancer cells secrete lysyl-tRNA synthetase (KRS) within exosomes. These KRS-containing exosomes induce inflammation and macrophage migration, potentially contributing to tumor progression (Kim et al., 2017).

Several models have been proposed to explain the mechanism of RNA molecule selection for release into EVs, in which RNA-binding proteins select RNA molecules through RNA-binding domains (Villarroya-Beltri et al., 2014; Santangelo et al., 2016; Shurtleff et al., 2017; Zietzer et al., 2018; Temoche-Diaz et al., 2019; Mosbach et al., 2021). Our findings on the presence of orthologous proteins in fungal EVs, such as tRNA synthetases, helicases, translation initiation, and elongation factors, suggest that there might be similar mechanisms for EV cargo packing among different fungal species. Our data provide a descriptive analysis of proteins related to RNA metabolism in EVs derived from pathogenic fungi. With the further molecular characterization of these proteins, clues to the RNA sorting mechanism into fungal and potentially other species’ EVs may be elucidated.

## Materials and methods

### Data collection and analysis

The data analyzed in this work was collected by the database that gathered EV proteomics from nine fungal species (Parreira et al., 2021). We selected the proteins annotated with the following terms: RNA-binding protein, RNA metabolism, translation, transcription, RNA helicase, and RRM. Next, we performed an alignment for the proteins with unknown functions using BLASTp to identify proteins with RNA-related roles (Altschul et al., 1997). We removed the ribosomal proteins from the analysis to avoid bias toward translation. We used the Uniprot and GeneDB databases to analyze function prediction and other features. For the proteins annotated as hypothetical or uncharacterized, we also searched for conserved domains at Uniprot, PFAM, and Interpro that could be associated with RNA metabolism. We also performed a protein BLAST analysis using the default parameters (Altschul et al., 1990).

### GO analysis

The GO term analysis was carried out in the database: FungiFun v. 2.2.8, available at: https://elbe.hki-jena.de/fungifun/. Using the parameters: significance test – hypergeometric distribution, test gene for category associations – over representation; adjustment method – Benjamimi-Hochberg procedure; annotation type – use Only directed annotated associations; GO advanced options – all; filter by evidence code – select all evidence codes. We then selected the terms with an FDR (Benjamimi-Hochberg) below 5%.

### Orthology

We used the CD-HIT v.4.8.1 software tool to cluster protein sequences from our dataset (Li and Godzik, 2006). The input file, containing all protein sequences, was formatted in FASTA, and clustering was performed with an identity threshold of 50%, a coverage cutoff of 80%, and a word size of 3. Finally, the cluster representative proteins were isolated using a custom Python script. The resulting non-redundant protein sequence set effectively represents the distinct clusters obtained from the clustering process.

## Data availability statement

The datasets presented in this study can be found in online repositories. The names of the repository/repositories and accession number(s) can be found in the article/**Supplementary Material**.

## Author contributions

MD performed the database search and analysis and wrote the manuscript. WKO performed the ortholog analysis. LA performed the analysis and helped to write the manuscript. MR and SG discussed all the results and contributed to writing the manuscript. All authors contributed to the article and approved the submitted version.
